# Draft Genome Sequence of the Nitrogen-Fixing Endophyte Azoarcus communis SWub3

**DOI:** 10.1128/MRA.01080-18

**Published:** 2018-10-04

**Authors:** Violeta Zorraquino, David Toubiana, Dawei Yan, Eduardo Blumwald

**Affiliations:** a Department of Plant Sciences, University of California, Davis, California, USA; Queens College

## Abstract

Here we report a draft genome sequence of Azoarcus communis SWub3, a nitrogen-fixing bacterium isolated from root tissues of Kallar grass in Pakistan.

## ANNOUNCEMENT

Biological nitrogen fixation is a process in which a living organism reduces atmospheric dinitrogen into two NH_3_ molecules. This reaction is catalyzed by the nitrogenase complex present exclusively in *Bacteria* and *Archaea* species. Plants can benefit from biological nitrogen fixation when they are in association with these nitrogen-fixing prokaryotes, either free living or as symbionts associated with their roots. Azoarcus is a bacterial genus that comprises species isolated from different environments, such as plant roots, sediments, aquifers, and contaminated soil (1–4). All Azoarcus species are Gram-negative rods with a strictly aerobic metabolism that can fix nitrogen microaerobically. The interest in this bacterial genus resides in its ability to efficiently infect several crops, including rice, which is a food staple for more than half of the world’s population (5–7). Moreover, one of its strains, Azoarcus sp. BH72, has been established as a model for nitrogen-fixing endophytes in grass (8). All plant-associated Azoarcus species share several features, like their ability to grow on various organic acids but not on carbohydrates, high optimum growth temperatures (37 to 42°C), and doubling times of 2 h (1).

Azoarcus communis SWub3 (=LMG 9095) was isolated from Kallar grass [*Leptochloa fusca* (L.) Kunth] roots in Punjab (Pakistan) in 1988 (1). For genomic sequencing, A. communis was grown in 4 ml of liquid ATCC medium 3 at 28°C. After 16 h, cells were harvested, and the DNA was extracted using the Wizard genomic DNA purification kit (Promega). The library was prepared using a KAPA HyperPrep kit (Roche) and run in a MiSeq PE300 cycle at the University of California, Davis, DNA Technologies Core. The run yielded 2,251,695 paired-end reads. Assemblies were done using SPAdes v. 3.12 with the careful option to minimize mismatches and provided an average of 192.3-fold coverage (9), as computed by the SPAdes software. The obtained genome sequence included 231 contigs (>200 bp) with an *N*_50_ value of 217,902 for contigs greater than 200. The calculated genome size was 5,004,685 bp with an average GC content of 62.5%. The genome sequence was annotated using the NCBI Prokaryotic Genome Annotation Pipeline (10). A total of 4,771 genes were predicted. The annotation includes 10 rRNA genes (5S, 16S, and 23S), 52 tRNA genes, and 4,428 protein-coding genes. Functional annotation with Rapid Annotations using Subsystems Technology (RAST) (11) showed that, among others, 179 genes were associated with motility and chemotaxis, 117 with nitrogen metabolism, 101 with virulence, disease, and defense, and 3 with dormancy and sporulation. Using the 16S rRNA gene sequence we found that its closest related type strain is Azoarcus sp. strain RS4 16S (GenBank accession number MH394448), a strain isolated from a sulfydogenic bioreactor in Bulgaria, with 98% homology.

This sequence allowed us to identify the nitrogenase operon and other loci involved in bacterium-plant symbiosis. It provides additional data to broaden our knowledge about the interaction of this nitrogen-fixing bacterial species with plants.

### Data availability.

The sequence data for Azoarcus communis SWub3 have been deposited in GenBank under the accession number GCA_003226565.
